# Supplementary material for: Bayesian analyses of radiocarbon dates suggest multiple origins of ceramic technology in Early Holocene Africa
Source: Nat Commun. 2025 Oct 3;16:8819. doi: 10.1038/s41467-025-63887-0 (PMC12494898; doi:10.1038/s41467-025-63887-0)
Supplement: Supplementary file 1 — Supplementary Information [file 41467_2025_63887_MOESM1_ESM.pdf]

Supplementary Information

Bayesian analyses of radiocarbon dates suggest multiple origins of ceramic technology in Early Holocene Africa.

Rocco Rotunno*[1,2], rotunno.rocco@gmail.com; rr695@cam.ac.uk

Enrico R. Crema[1,3], erc62@cam.ac.uk

[1] McDonald Institute for Archaeological Research, University of Cambridge, UK
[2] The Archaeological Mission in the Sahara, Sapienza University of Rome, IT
[3] Department of Archaeology, University of Cambridge, UK

*corresponding author

Supplementary Note 1: Data Preparation

Our initial dataset consisted of 855 radiocarbon dates from 259 sites. After calibrating each sample using the IntCal20 calibration curve (*1*), we used the *binPrep* function of the *rcarbon* R package (*2*), setting *h* to 100 years to identify clusters of dates close in time that can be considered from the same occupation. We then used the *thinDates* to randomly select one sample from each bin, prioritising dates with smaller ^{14}C errors. In some rare cases, dates from the same bin were inconsistently associated with the presence/absence of ceramic. For those instances, we assumed the ceramic to be present and hence assigned the presence of ceramic to the randomly sampled date. The final sample size consisted of 587 radiocarbon dates. As we considered only presence data for the quantile regression model, our sample was reduced to 338.

Supplementary Note 2: Model Definition

2.1 Quantile Regression Model Definition

$$\theta_i \sim \text{AsymLaplace}(\mu_i, \lambda, \tau)$$
$$\tau = 0.95$$
$$\mu_i = \gamma_0 + \gamma_1 d_{o,i}$$
$$x_i \sim \text{Normal}(f_1(\theta_i), \sigma_i)$$
$$\sigma_i = (\varepsilon_i^2 + f_2(\theta_i)^2)^{0.5}$$
$$\gamma_0 \sim \text{TruncatedNormal}(11000, 1000, 500, 50000)$$
$$\gamma_1 \sim \text{Normal}(0,1)$$
$$\tau \sim \text{Exponential}(0.05)$$

Where:

- θ_i Is the calendar date of sample *i* in BP
- x_i Is the ^{14}C age of sample *i*
- ε_i Is the ^{14}C age error of sample *i*
- $d_{o,i}$ Is the distance of sample *i* from the putative point of origin *o*.
- $f_1(\theta_i)$ Is the ^{14}C age matching the calendar date θ_i on the IntCal20 calibration curve.
- $f_2(\theta_i)$ Is the ^{14}C age error matching the calendar date θ_i on the IntCal20 calibration curve.

2.2 Binomial Regression Model Definition

Single Origin (models m_1, m_2, and m_3)

$$
\begin{gathered}
y_i \sim \text{Bernoulli}(p_i) \\
\text{logit}(p_i) = \beta_0 - \beta_1 \theta_i - \beta_2 d_{1,i} + \beta_3 \theta_i d_{1,i} \\
\theta_i \sim \text{Uniform}(1000, 30000) \\
x_i \sim \text{Normal}(f_1(\theta_i), \sigma_i) \\
\sigma_i = (\varepsilon_i^2 + f_2(\theta_i)^2)^{0.5} \\
\beta_0 \sim \text{Normal}(0, 0.5) \\
\beta_1 \sim \text{TruncatedNormal}(0, 0.5, 0, \infty) \\
\beta_2 \sim \text{TruncatedNormal}(0, 0.5, 0, \infty) \\
\beta_3 \sim \text{Normal}(0, 0.5)
\end{gathered}
$$

Dual Origin (models m_4,m_5, and m_6)

$$
\begin{gathered}
y_i \sim \text{Bernoulli}(p_{i,max}) \\
p_{i,max} = \max(p_{i,1}, p_{i,2}) \\
\text{logit}(p_{i,1}) = \beta_{0,1} - \beta_{1,1} \theta_i - \beta_{2,1} d_{1,i} + \beta_{3,1} \theta_i d_{1,i} \\
\text{logit}(p_{i,2}) = \beta_{0,2} - \beta_{1,2} \theta_i - \beta_{2,2} d_{2,i} + \beta_{3,2} \theta_i d_{2,i} \\
\theta_i \sim \text{Uniform}(1000, 30000) \\
x_i \sim \text{Normal}(f_1(\theta_i), \sigma_i) \\
\sigma_i = (\varepsilon_i^2 + f_2(\theta_i)^2)^{0.5} \\
\beta_{0,1} \sim \text{Normal}(0, 0.5) \\
\beta_{0,2} \sim \text{Normal}(0, 0.5)
\end{gathered}
$$

$$
\begin{gathered}
\beta_{1,1} \sim \text{TruncatedNormal}(0, 0.5, 0, \infty) \\
\beta_{1,2} \sim \text{TruncatedNormal}(0, 0.5, 0, \infty) \\
\beta_{2,1} \sim \text{TruncatedNormal}(0, 0.5, 0, \infty) \\
\beta_{2,2} \sim \text{TruncatedNormal}(0, 0.5, 0, \infty) \\
\beta_{3,1} \sim \text{Normal}(0, 0.5) \\
\beta_{3,2} \sim \text{Normal}(0, 0.5)
\end{gathered}
$$

Triple Origin (model m_7)

$$y_i \sim \text{Bernoulli}(p_{i,max})$$
$$p_{i,max} = \max(p_{i,1}, p_{i,2}, p_{i,3})$$
$$\text{logit}(p_{i,1}) = \beta_{0,1} - \beta_{1,1}\,\theta_i - \beta_{2,1}\,d_{1,i} + \beta_{3,1}\,\theta_i\,d_{1,i}$$
$$\text{logit}(p_{i,2}) = \beta_{0,2} - \beta_{1,2}\,\theta_i - \beta_{2,2}\,d_{2,i} + \beta_{3,2}\,\theta_i\,d_{2,i}$$
$$\text{logit}(p_{i,3}) = \beta_{0,3} - \beta_{1,3}\,\theta_i - \beta_{2,3}\,d_{3,i} + \beta_{3,3}\,\theta_i\,d_{3,i}$$
$$\theta_i \sim \text{Uniform}(1000, 30000)$$
$$x_i \sim \text{Normal}(f_1(\theta_i), \sigma_i)$$
$$\sigma_i = (\varepsilon_i^2 + f_2(\theta_i)^2)^{0.5}$$
$$\beta_{0,1} \sim \text{Normal}(0, 0.5)$$
$$\beta_{0,2} \sim \text{Normal}(0, 0.5)$$
$$\beta_{0,3} \sim \text{Normal}(0, 0.5)$$
$$\beta_{1,1} \sim \text{TruncatedNormal}(0, 0.5, 0, \infty)$$
$$\beta_{1,2} \sim \text{TruncatedNormal}(0, 0.5, 0, \infty)$$
$$\beta_{1,3} \sim \text{TruncatedNormal}(0, 0.5, 0, \infty)$$
$$\beta_{2,1} \sim \text{TruncatedNormal}(0, 0.5, 0, \infty)$$
$$\beta_{2,2} \sim \text{TruncatedNormal}(0, 0.5, 0, \infty)$$
$$\beta_{2,3} \sim \text{TruncatedNormal}(0, 0.5, 0, \infty)$$
$$\beta_{3,1} \sim \text{Normal}(0, 0.5)$$
$$\beta_{3,2} \sim \text{Normal}(0, 0.5)$$
$$\beta_{3,3} \sim \text{Normal}(0, 0.5)$$

Where:

- θ_i Is the calendar date of sample *i* in BP
- x_i Is the ^{14}C age of sample *i*
- ε_i Is the ^{14}C age error of sample *i*
- $d_{1,i}$ Is the distance of sample *i* from the putative point of origin 1, $d_{2,i}$ is the distance from origin 2, and $d_{3,i}$ is the distance from origin 3.

2.3 Stratigraphic Constraints for θ

When stratigraphic information was available, we further defined constraints for the values of θ. For example, if sample *i* from a given site was thought to be from an earlier context to sample *j* from the same site, we would add the constraint $\theta_i > \theta_j$. We implemented 34 constraints for the quantile regression model and 41 for the binomial model.

2.4 Parameter Scaling

To facilitate MCMC convergence in the binomial models, we scaled both θ and d using the observed mean and standard deviations of the calibrated median ages for the former.

Supplementary Note 3: MCMC Settings, Diagnostics, and Posteriors

3.1 MCMC Settings

All models have been fitted via MCMC using the *nimble (3, 4)* and the *nimbleCarbon* (*5*, *6*) R packages. All ten models (i.e. the three quantile regression models and the seven binomial models) were fitted over four chains. Quantile regression models were fitted using 100,000 chains, half discarded during the burn-in phase, with posterior samples collected every 5 steps. Binomial models required slightly longer chains, with 500,000 iterations, 250,000 used for burn-in, and samples taken every 25 steps. We used default samplers suggested by the nimble package for the quantile regression models (*RW_sampler* for all parameters). In contrast, for models m_4 - m_7 in the binomial regression, we used *AF_sampler* with custom tuning settings for all the β parameters while we kept the default RW_sampler for θ. This adjustment was necessary to control for the inevitably high correlation between the β when multiple points of origin were considered. Details of the MCMC settings can be found in the relevant scripts `prepare_quantref_02b.R` and `prepare_binom_02a.R` on the online repository at https://github.com/roccorot/OriginPotteryNAfrica.

3.2 Diagnostics and Posteriors

We evaluated the performance of the MCMC using the Gelman-Rubin statistic (R-hat; REF) and effective sample size. All models yielded good convergence (R-hat < 1.01) and good sampling efficiency (ESS > 10,000). Supplementary Tab.1 and Supplementary Data 1 show the posterior mean, the 95% highest posterior density interval (HPDI), the Gelman-Rubin R-hat diagnostic, and the effective sample size for all key parameters (i.e. excluding θ for our two sets of models).

Origin	Parameters	Posterior Mean	95% HPDI	R-hat	ESS
Bir Kiseiba	γ_0	10,175 BP	10,044 ~ 10,297 BP	1.001	7131
	γ_1	0.0000804	0.0000062 ~ 0.0001562	1.001	8574
Adrar Bous 10	γ_0	10,285 BP	10,115 ~ 10,456 BP	1.001	3558
	γ_1	-0.0000129	-0.0001063 ~ 0.0000822	1.002	3625
Ounjougou Ravin de la Mouche	γ_0	10,617 BP	10,414 ~ 10,819 BP	1.001	2965
	γ_1	-0.0001355	-0.0002061 ~ -0.0000626	1.001	2719

Supplementary Table 1. Posteriors and MCMC Diagnostics for the three quantile regression models.

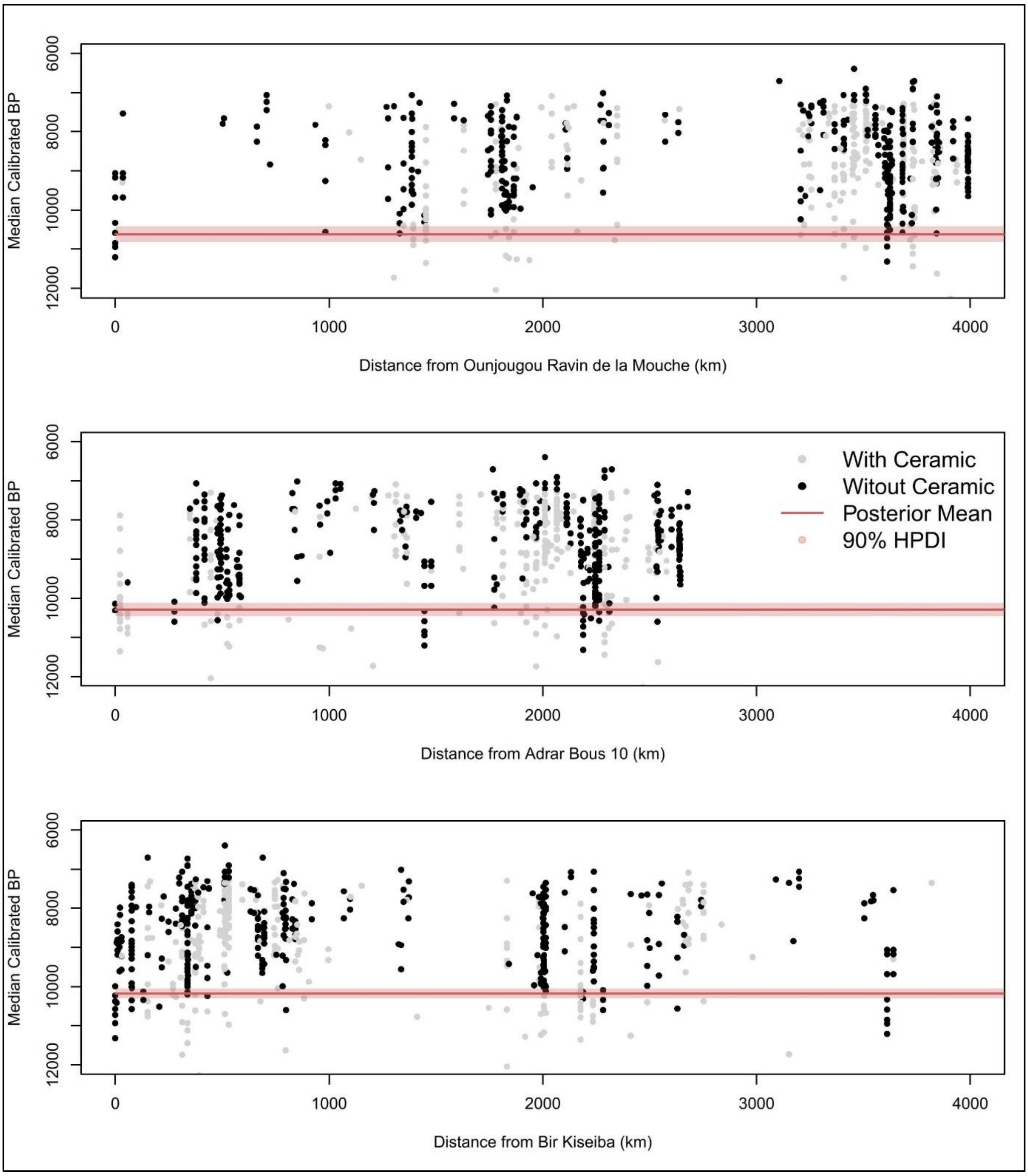

Supplementary Figure 1. **Quantile regression models for the three putative points of origin.** Median calibrated radiocarbon ages (cal BP) plotted against distance from three reference sites: Ounjougou Ravin de la Mouche (top), Adrar Bous 10 (middle), and Bir Kiseiba (bottom). Black points represent sites with ceramics, while grey points represent sites without ceramics. The red line indicates the posterior mean estimate, with the shaded band showing the 90% highest posterior density interval (HPDI).

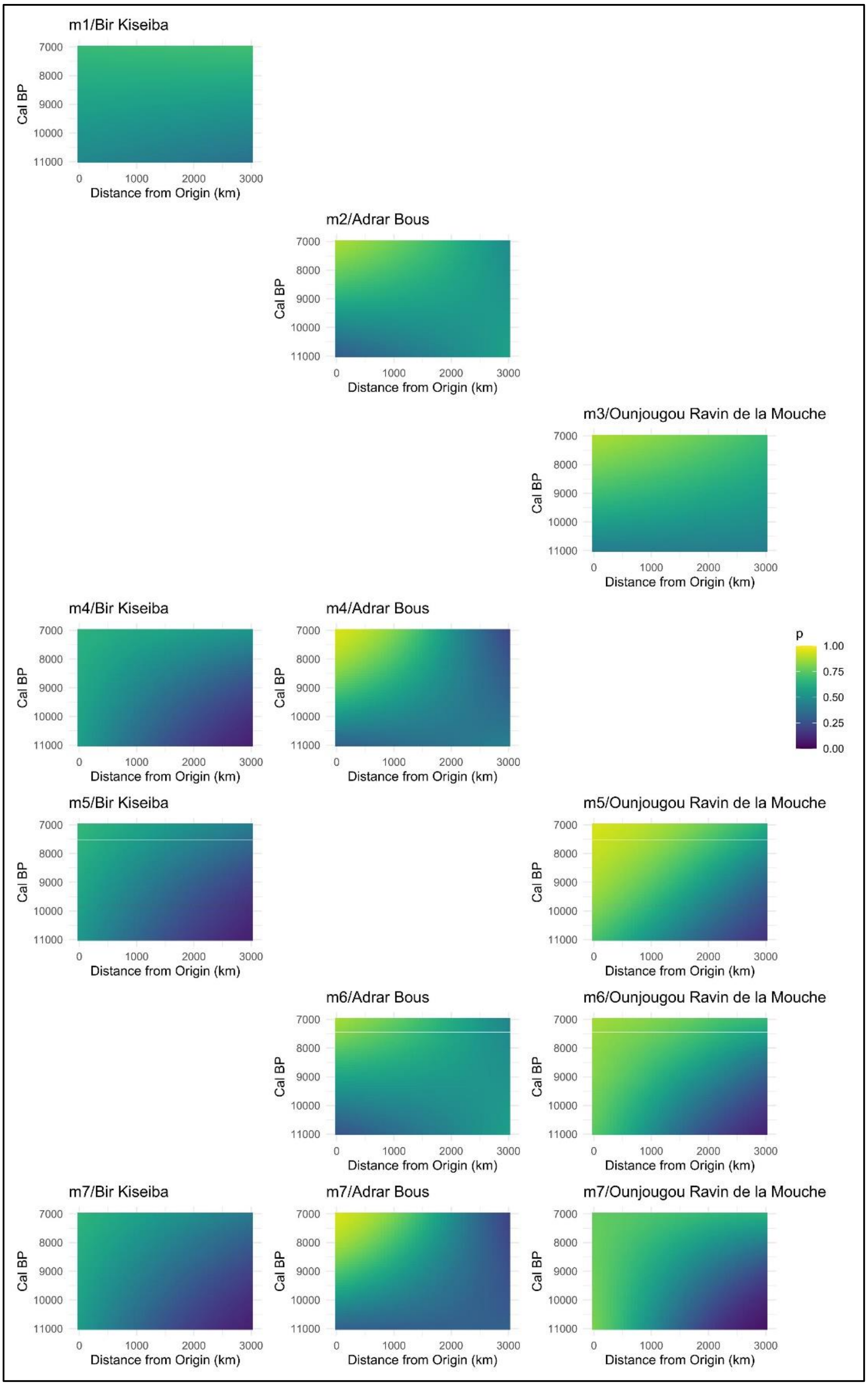

Supplementary Figure 2. **Posterior probability of ceramic occurrence (m1–m7)**. Marginal posterior mean probability of ceramic as a function of distance from origin (Km) and time (cal BP) for the seven binomial models (m1-m7). Results are shown for three potential origin points: Bir Kiseiba, Adrar Bous, and Ounjougou Ravin de la Mouche. The color scale indicates posterior probability (P), from low (purple) to high (yellow).

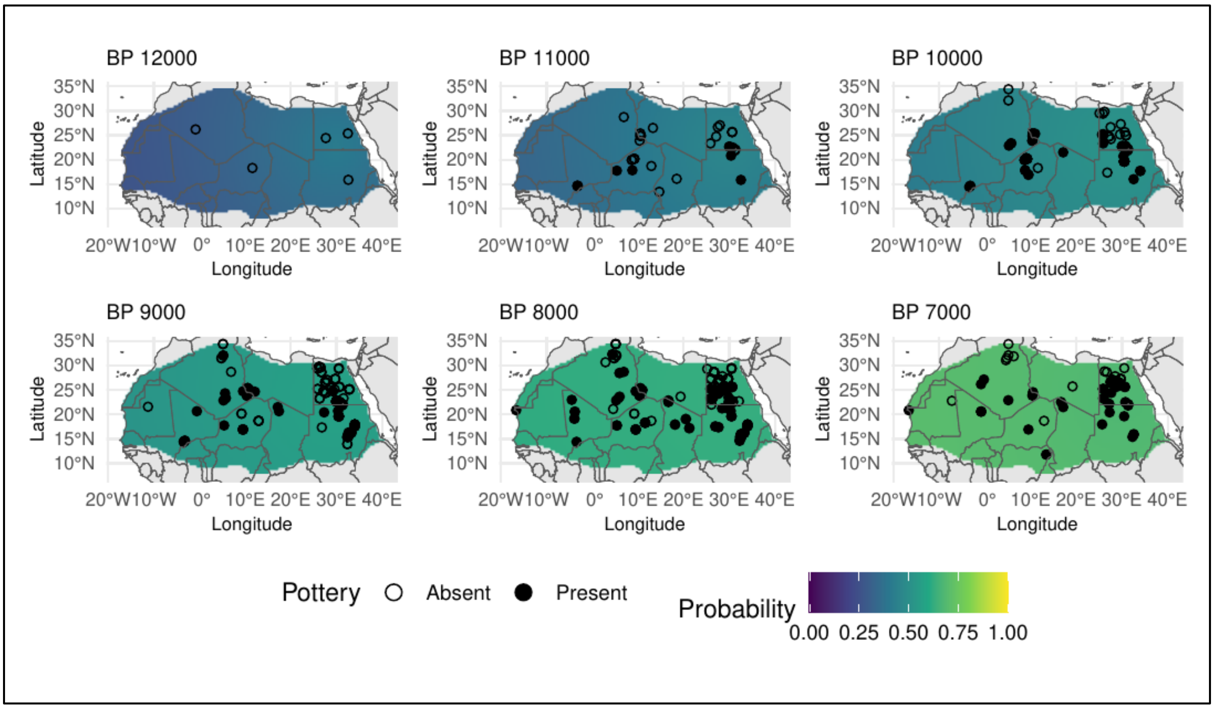

Supplementary Figure 3. **Posterior mean probability for the presence of ceramic for model m_1.** The filled points and circles show the locations of the samples with a cumulative calibrated probability mass over 0.5 for the interval spanning 500 years before and after each time slice.

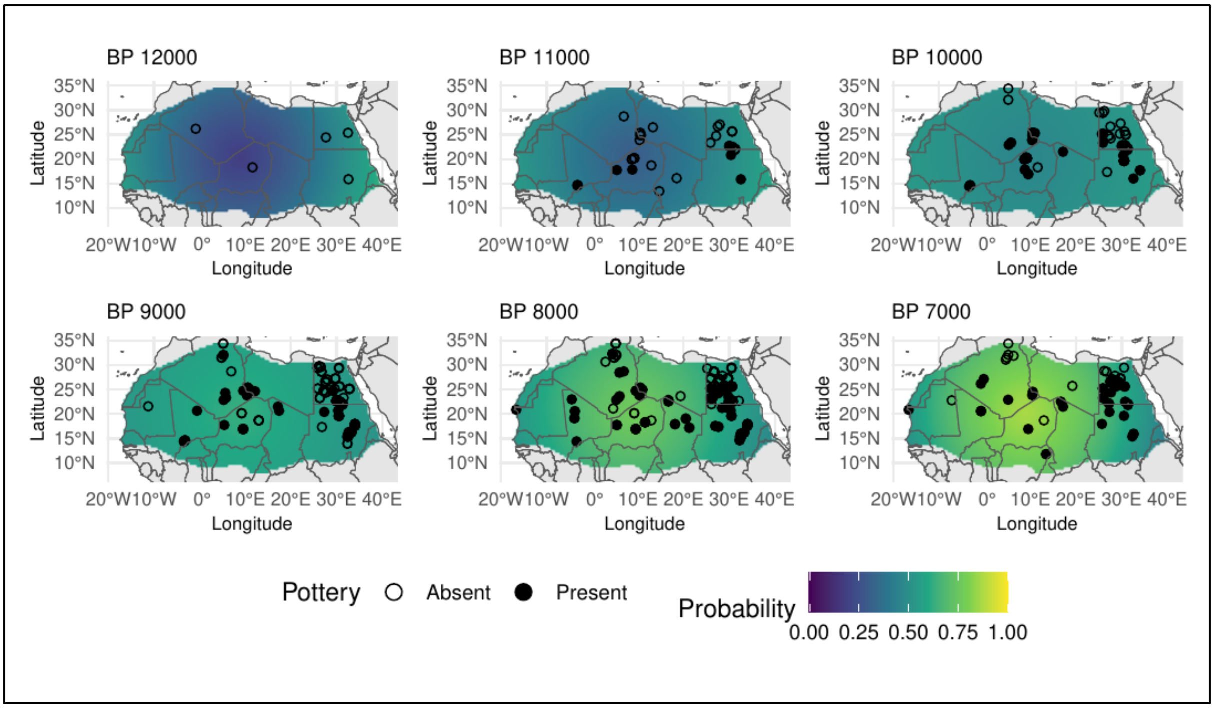

Supplementary Figure 4. **Posterior mean probability for the presence of ceramic for model m_2.** The filled points and circles show the locations of the samples with a cumulative calibrated probability mass over 0.5 for the interval spanning 500 years before and after each time slice.

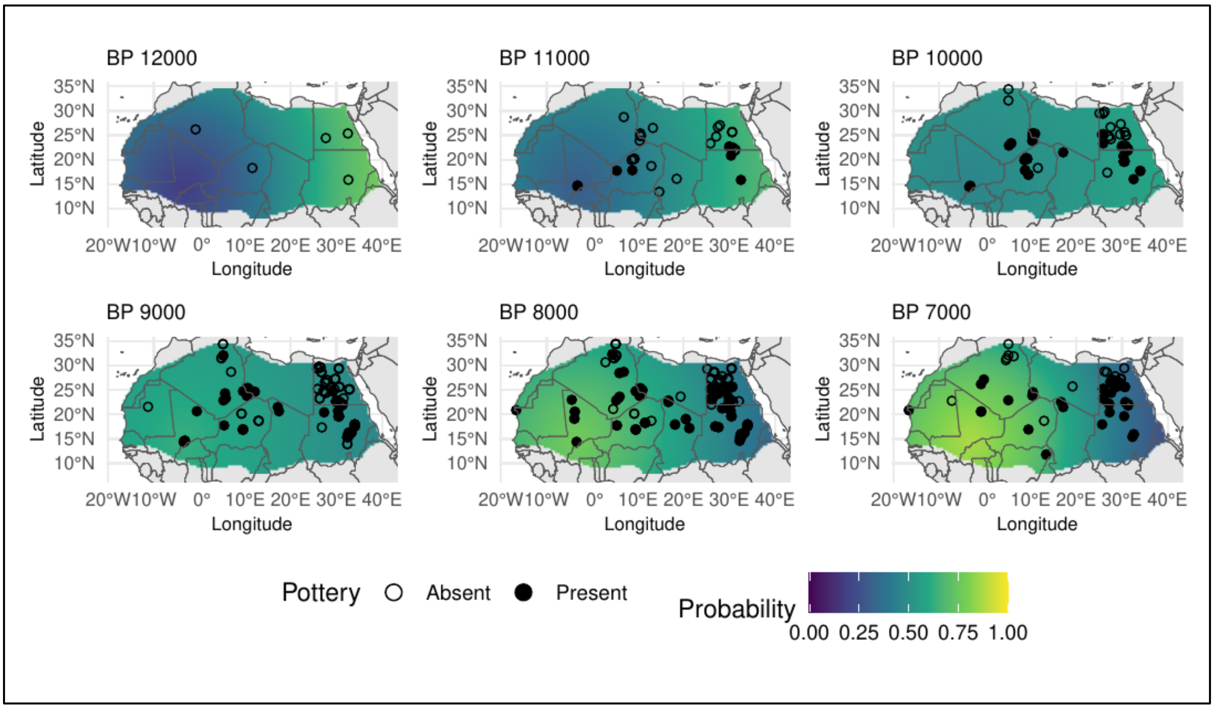

Supplementary Figure 5. **Posterior mean probability for the presence of ceramic for model m_3.** The filled points and circles show the locations of the samples with a cumulative calibrated probability mass over 0.5 for the interval spanning 500 years before and after each time slice.

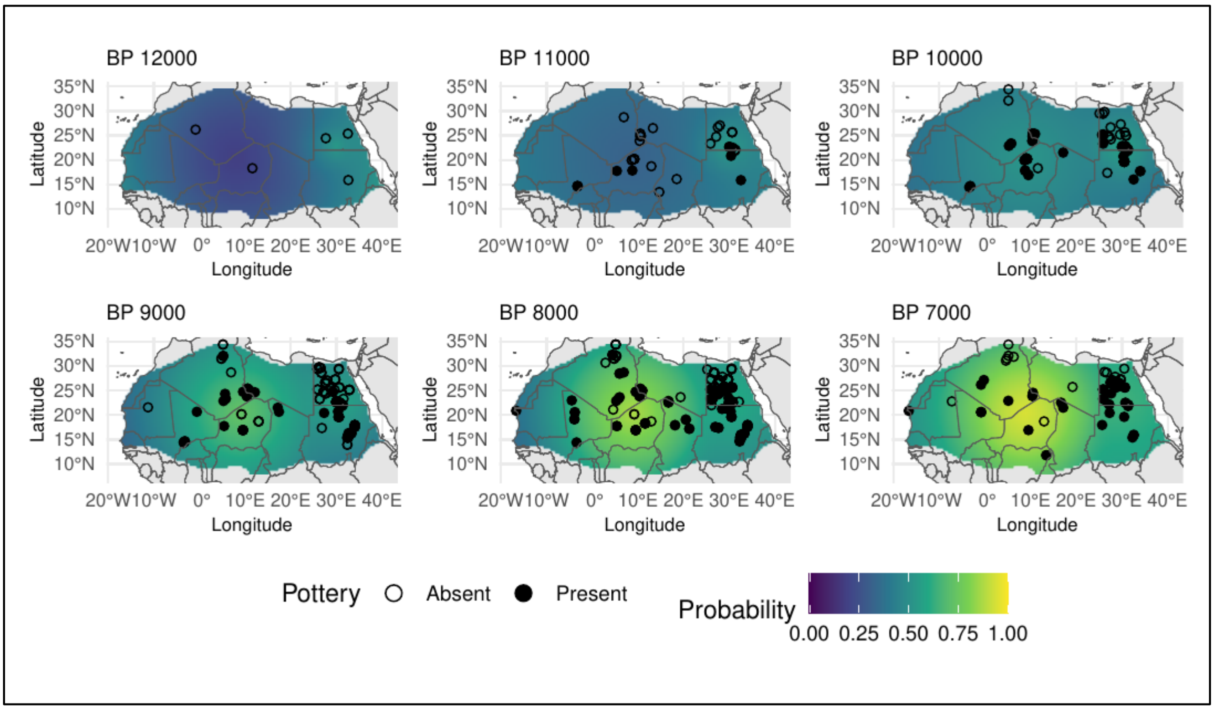

Supplementary Figure 6. **Posterior mean probability for the presence of ceramic for model m_4.** The filled points and circles show the locations of the samples with a cumulative calibrated probability mass over 0.5 for the interval spanning 500 years before and after each time slice.

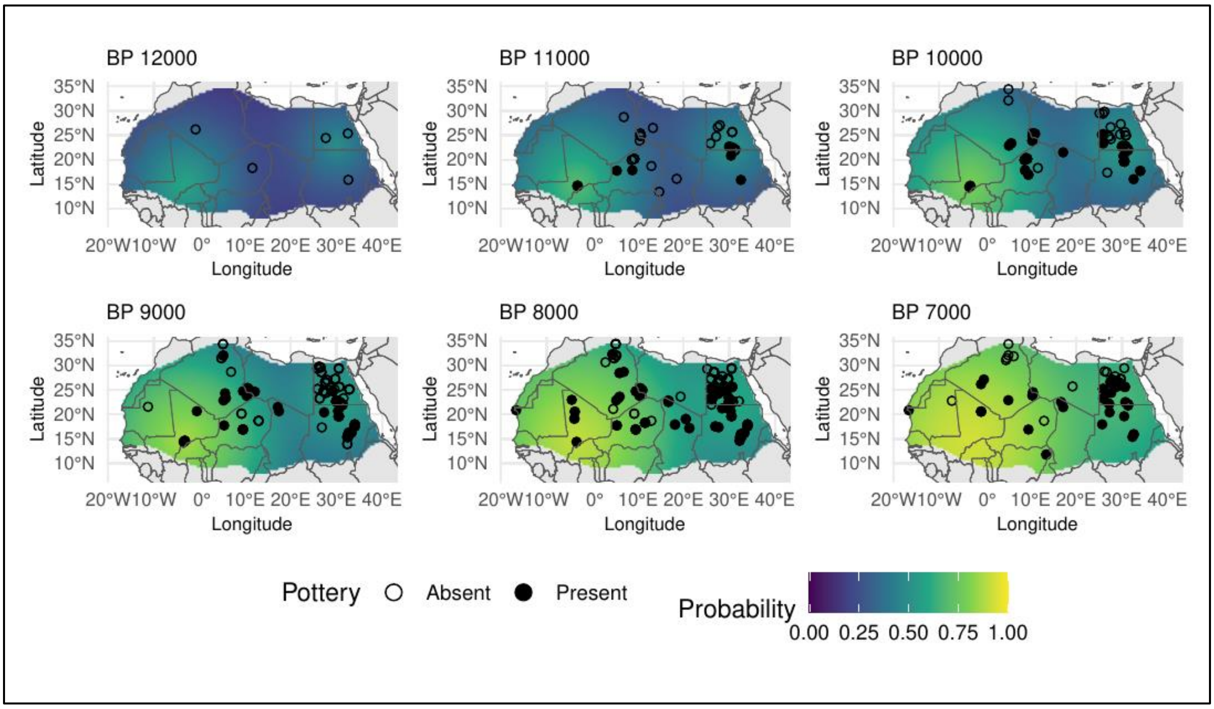

Supplementary Figure 7. **Posterior mean probability for the presence of ceramic for model m_5**. The filled points and circles show the locations of the samples with a cumulative calibrated probability mass over 0.5 for the interval spanning 500 years before and after each time slice.

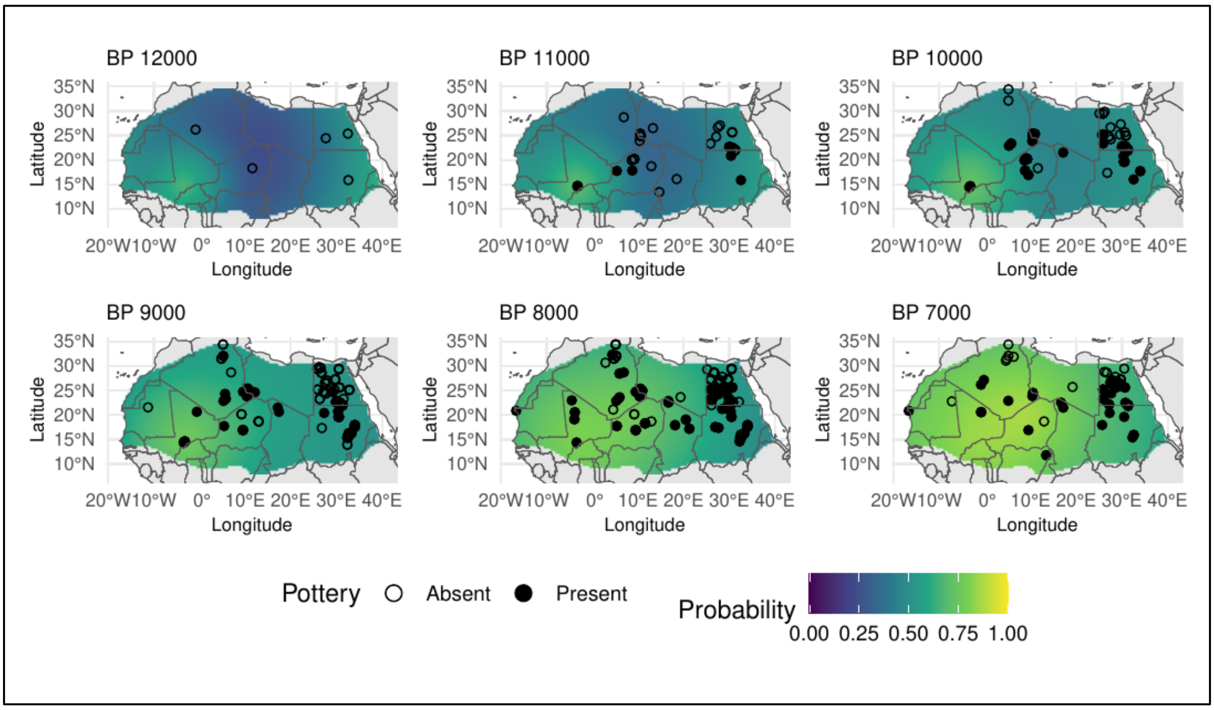

Supplementary Figure 8. **Posterior mean probability for the presence of ceramic for model m_6**. The filled points and circles show the locations of the samples with a cumulative calibrated probability mass over 0.5 for the interval spanning 500 years before and after each time slice.

3.3 Spatial Analyses of Residuals

We further examined whether and where the assumption of an isotropic diffusion is invalid by examining the spatial distribution of the model residuals for the model with the lowest WAIC (m_7). We computed model residuals r_i for each location i as the difference between our binary observations (y_i) and the median posterior estimate of $p_{i,max}$, i.e. the expected probability of the presence of ceramic. The potential presence of *cold spots* (i.e. significant clustering of lower values of r_i, e.g. areas with a concentration of sites with ceramic absence where the model expected presence), and *hot spots* (i.e. significant clustering of high values of r_i, e.g. areas with a concentration of sites with the presence of ceramic absence where the model expected lower probability of presence) were evaluated using local Getis-Ord statistic (*7*). We conducted the analyses via the *sfdep* R package (*8*) for selected time slices with a larger number of sample observations (10k BP, 9k BP, 8kBP, and 7kBP), including all observations with a cumulative calibrated probability mass equal to or larger than 0.5 and considering a neighbourhood distance of 1,000 km.

Supplementary references

1. P. J. Reimer, W. E. N. Austin, E. Bard, A. Bayliss, P. G. Blackwell, C. B. Ramsey, M. Butzin, H. Cheng, R. L. Edwards, M. Friedrich, P. M. Grootes, T. P. Guilderson, I. Hajdas, T. J. Heaton, A. G. Hogg, K. A. Hughen, B. Kromer, S. W. Manning, R. Muscheler, J. G. Palmer, C. Pearson, J. van der Plicht, R. W. Reimer, D. A. Richards, E. M. Scott, J. R. Southon, C. S. M. Turney, L. Wacker, F. Adolphi, U. Büntgen, M. Capano, S. M. Fahrni, A. Fogtmann-Schulz, R. Friedrich, P. Köhler, S. Kudsk, F. Miyake, J. Olsen, F. Reinig, M. Sakamoto, A. Sookdeo, S. Talamo, The IntCal20 Northern Hemisphere Radiocarbon Age Calibration Curve (0–55 cal kBP). *Radiocarbon* **62**, 725–757 (2020).
2. E. R. Crema, A. Bevan, Inference from large sets of radiocarbon dates: software and methods. *Radiocarbon* **63**, 23–39 (2021).
3. P. de Valpine, D. Turek, C. J. Paciorek, C. Anderson-Bergman, D. T. Lang, R. Bodik, Programming With Models: Writing Statistical Algorithms for General Model Structures With NIMBLE. *J. Comput. Graph. Stat.* **26**, 403–413 (2017).
4. P. de Valpine, C. Adler, D. Turek, N. Michaud, C. Anderson-Bergman, F. Obermeyer, C. Wehrhahn Cortes, A. Rodrìguez, D. Temple Lang, S. Paganin, *NIMBLE: MCMC, Particle Filtering, and Programmable Hierarchical Modeling* (2020; https://doi.org/10.5281/zenodo.1211190).
5. E. R. Crema, S. Shoda, A Bayesian approach for fitting and comparing demographic growth models of radiocarbon dates: A case study on the Jomon-Yayoi transition in Kyushu (Japan). *PLoS One* **16**, e0251695 (2021).
6. E. R. Crema, nimbleCarbon: Models and Utility Functions for Bayesian Analyses of Radiocarbon Dates with NIMBLE (v.0.2.0). [Preprint] (2022). https://github.com/ercrema/nimbleCarbon.
7. A. Getis, J. K. Ord, The analysis of spatial association by use of distance statistics. *Geogr. Anal.* **24**, 189–206 (1992).
8. J. Parry, D. Locke, *Sfdep: Spatial Dependence for Simple Features* (2024; https://CRAN.R=project.org/package=sfdep).
