# Supplementary material for: Bayesian analyses of radiocarbon dates suggest multiple origins of ceramic technology in Early Holocene Africa
Source: Nat Commun. 2025 Oct 3;16:8819. doi: 10.1038/s41467-025-63887-0 (PMC12494898; doi:10.1038/s41467-025-63887-0)
Supplement: Supplementary file 2 — Description of Additional Supplementary File [file 41467_2025_63887_MOESM2_ESM.pdf]

Description of Additional Supplementary File

Supplementary Data 1

Posterior estimates and Markov Chain Monte Carlo (MCMC) diagnostics for the binomial regression models (m1–m7). The dataset reports the posterior mean, 95% highest posterior density interval (HPDI), Gelman–Rubin convergence statistic (R-hat), and effective sample size (ESS) for each parameter ($\beta 0$–$\beta 3$) across the three putative points of origin: Bir Kiseiba (b), Adrar Bous 10 (a), and Ounjougou Ravin de la Mouche (o).
